# Development of an Empirical Model as a Prediction Tool for the Sound Absorption Performance of Wool/Soy Protein Biocomposites

**DOI:** 10.3390/polym17192666

**Published:** 2025-10-02

**Authors:** Jesús Alba, Marta Urdanpilleta, Romina del Rey, Itsaso Leceta, Pedro Guerrero, Koro de la Caba

**Affiliations:** 1 Centre for Physics Technologies: Acoustics, Materials and Astrophysics, EPS Gandia, Universitat Politècnica de València, C/Paraninf, 1, 46730 Grao de Gandia, Spain; jesalba@fis.upv.es (J.A.); roderey@fis.upv.es (R.d.R.); 2 Biomat Research Group, Escuela de Ingeniería de Gipuzkoa, University of the Basque Country (UPV/EHU), Plaza de Europa 1, 20018 Donostia-San Sebastián, Spain; marta.urdanpilleta@ehu.eus (M.U.); itsaso.leceta@ehu.eus (I.L.)

**Keywords:** biocomposites, sheep wool, sound insulation, airflow resistivity, sound absorption

## Abstract

Finding eco-friendly alternatives to the synthetic materials used for acoustic application in building industry is necessary to address environmental sustainability. Biocomposites of natural fibers combined with a biopolymer matrix emerge as a promising approach. In this study, soy protein biocomposites were prepared with 10, 15, and 20 wt% sheep wool and were added spent coffee grounds by freeze-drying to create fibro-porous biocomposites for acoustic applications. Transmission loss (*TL*) measurements underlined good behavior as sound insulators, with maximum values around 22 dB at 2500 Hz and even better performance than those of commercial synthetic solutions. The obtained sound absorption coefficients were competitive, as they almost reached unity at medium and high frequencies. Airflow resistivity was determined, and values were higher for the biocomposites with coffee grounds, specifically 14–18 kPa·s·m^−2^ vs. 5.62–11.6 kPa·s·m^−2^. Using the input of the measured airflow resistivity, an empirical model using a genetic algorithm was developed as a prediction tool for the sound absorption performance of the samples. All in all, results showcase the feasibility of employing the studied biocomposites as competitive sound insulators and absorbers in building construction industry.

## 1. Introduction

The construction industry is among the largest consumers of energy and raw materials worldwide [1,2]. Within the EU, it contributes to nearly 40% of emissions and accounts for almost a third of all waste generated. Only around 40% of construction waste undergoes recycling or reuse during building demolition [3]. In this context, the 2030 Agenda [4] includes within its goals the need for sustainable cities and communities (11th goal) while ensuring good health and well-being (3rd goal). Therefore, there is an interest in obtaining appropriate noise insulation and noise control, as today noise is a major pollutant that interferes in human activities and causes innumerable health problems.

If the focus is cast on sound and thermal insulating materials for building industry, glass wool and stone wool account for 60% of the European market [5]. Yet, from a circular economy point of view, there is no current closed loop solution to treat and recycle the glass wool waste generated during construction and/or demolition/deconstruction activities [6]. This waste of glass wool, if not reused on-site for little added value use like filling gaps, for instance, is most times landfilled [7]. In an evaluation of insulation materials according to environmental criteria that consider production and use, glass wool and stone wool are in a medium–high position of impact, mainly because of their production process [5].

Regarding a sustainable approach, secondary raw materials are materials recovered from waste and end-of-life products by recycling. They constitute a pivotal axis in the circular economy strategy [8,9], as they allow closing the loop and continuing the cycles of production and usage. In particular, sheep wool waste could develop into a useful secondary raw material. Nowadays, they still constitute a problem to be addressed by many local producers all around the world, because less and less breeds are attractive for being exploded in the textile market [10,11]. This business, which was lucrative in the past, has decayed due to the new consumption trends and the massive introduction of synthetic alternatives. Because of the polluting effect in soil or air of wool residues [10,12,13], farmers are forced to pay expenses for their sheep waste to be retired and treated. At the same time, although usage of wool that is not related to the textile industry has been studied [12,14,15,16], wool use is not extended and landfilling and incineration are still the conventional treating methods [12].

Trying to tackle those problems, performance and competitiveness of biocomposites of sheep wool with soy protein for application in building acoustics were evaluated in several works. First, by the determination of sound absorption coefficient and airflow resistivity [17,18] for samples with thicknesses typical of solid panels in double walls. This analysis revealed a performance that can compete with other natural origin alternatives and even synthetic commercial materials. Subsequently, spent coffee grounds were added to the formulation to increase the resistance against insects such as carpet beetle and clothe moth, and thermal behavior and sound absorption properties of the resulting biocomposites were evaluated [19]. This point is important, as materials sandwiched in building walls need to work in both types of insulation. Also, an empirical model for the prediction of sound absorption coefficient was developed based on the Delany–Bazley model, using an iterative method. Coffee grounds efficiently preserved biocomposites against the attack of clothe moth and carpet beetle larvae, while thermal properties were well within the commercial standards.

In this work, biocomposites with spent coffee grounds have been subjected to further acoustic characterization, through the measurement of *TL*, which is the key parameter for characterizing at lab scale the sound insulation performance of a material. Additionally, an empirical model based on a genetic algorithm as a different approach has been used as a predicting tool for the sound absorption of the biocomposites with coffee grounds. To develop and assess the suitability of the model, sound absorption coefficient and airflow resistivity have also been measured. As a result, sheep wool and coffee grounds residues as well as soya oil production by-products have been valorized and re-introduced in the circular economy chain, and it has been demonstrated that they constitute a feasible sustainable alternative to be used for acoustic applications in building construction.

## 2. Materials and Methods

### 2.1. Materials

Raw sheep wool of Latxa breed, characteristic of the Basque Country and Navarre (northern Spain), was provided by a local shepherdess (Ametzaga de Zuia, Araba, Spain). Soy protein isolate (SPI, PROFAM 974) was used as a polymeric binder, and it was purchased from ADM Protein Specialities Division (Amsterdam, The Netherlands). Glycerol (99.5% purity), supplied by Panreac (Barcelona, Spain), was used as a plasticizer. Spent coffee grounds (*C. arabica*) were locally achieved and used as an insecticidal additive.

### 2.2. Sample Preparation

**Figure 1:**
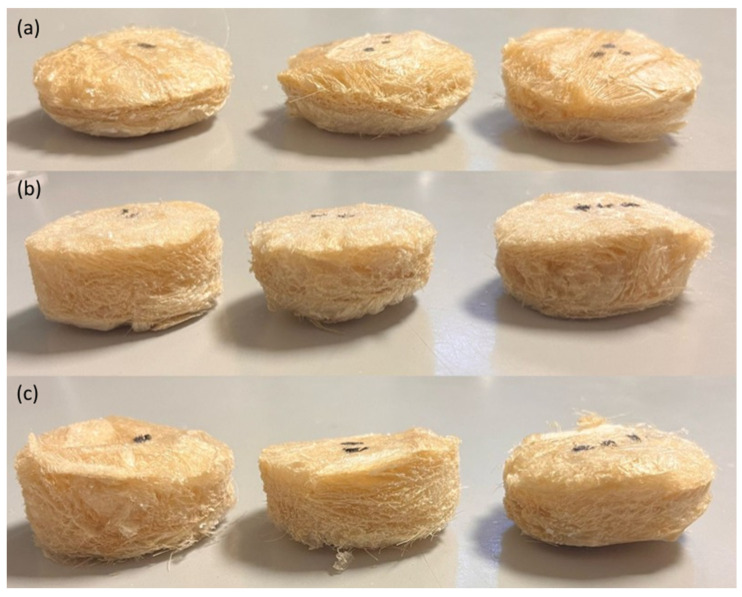
Images of soy protein isolate (SPI) biocomposites prepared with spent coffee grounds (C) by freeze-drying with a sheep wool content (W) of (**a**) 10, (**b**) 15, and (**c**) 20 wt%.

Raw wool was washed and dried following the procedure described in a previous work [17]. Raw wool was poured in water at 45 °C for 15 min, then removed. This process was repeated five times to remove dirt and lanoline. Then, wool was left to dry and stored in a chamber at 25 °C and 50% relative humidity until the preparation of the biocomposite. For that, 15 g of SPI, 4 wt% spent coffee grounds (C), and the corresponding amount of sheep wool (W) to achieve 10, 15, or 20 wt% (both C and W based on SPI dry basis) were mixed with 500 mL of distilled water and heated to 80 °C for 30 min under continuous stirring. Subsequently, 20 wt% glycerol (based on the dry weight of SPI) was added, and the pH was adjusted to 10 using 1 M NaOH. The resulting dispersions were maintained at 80 °C for additional 30 min while stirring at 150 rpm. At the end, dispersions were poured into molds and freeze-dried (Alpha 1–4 LD freeze-dryer Martin Chirst, Thermo Fisher, Waltham, MA, USA) to obtain the biocomposites, named SPIW10C, SPIW15C, and SPIW20C as a function of sheep wool content, as shown in Figure 1.

### 2.3. Measurements of Transmission Loss (TL)

**Figure 2:**
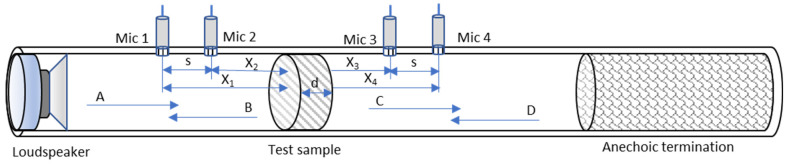
Device for measuring transmission loss (*TL*). A couple of microphones are placed on each side of the test sample.

*TL* is determined by means of impedance tubes according to the ASTM E2611—19 standard [20]. Figure 2 depicts a scheme of the measurement device.

The loudspeaker is placed at the end of the tube and generates plane waves. Two of the microphones are located in the tube between the speaker and the sample, and other two are placed at the rear end, between the sample and an anechoic termination. The device symbolizes the description of a transfer matrix of the incident and reflected waves of the upper and lower paths. In case the matrix coefficients are known, it is possible to obtain *TL* according to the following equation [20]:

(1) $$TL=20\log_{10}\left|\frac{e^{jks}-H_{12}}{e^{jks}-H_{34}}\right|-20\log_{10}\left|H_{t}\right|$$

where *s* is the distance between microphones, *k* is the wavenumber, *H*_12_ and *H*_34_ represent the transfer function between microphones 1 and 2 (pre-sample), and 3 and 4 (post-sample), respectively. The relationship between the autospectra and *H_t_* is defined by the equation [20]:

(2) $$H_{t}=\sqrt{\left|S_{d}/S_{u}\right|}$$

where *S_u_* is the autospectrum that precedes the sample, and *S_d_* is the autospectrum after the sample.

### 2.4. Measurements of Sound Absorption Coefficient

**Figure 3:**
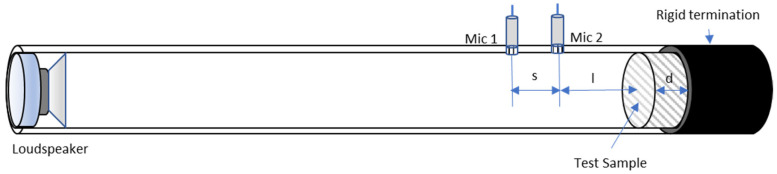
Scheme of the experimental arrangement (impedance tube or Kundt’s tube) used to measure the sound absorption coefficient. Both microphones are mounted at the same side of the sample tested.

Sound absorption coefficient at normal incidence was measured using the two-microphone transfer function technique and an impedance tube (Kund’s tube), according to ISO 10534-2:2024 [21]. In the transfer function method, a broadband stationary random signal is used, and the sound field is decomposed into incident and reflected waves [22]. The standing wave tube had an inner diameter of 40 mm. Two ½-inch laboratory-grade microphones (Bruel & Kjaer 4189, Madrid, Spain), connected to their respective preamplifiers (Bruel & Kjaer ZC0032, Madrid, Spain), were mounted flush with the tube walls. To allow obtaining measurements in the 125–3150 Hz frequency range, a distance between the microphones of 32 mm was adjusted. A loudspeaker (Beyma CP800TI, Valencia, Spain) was placed at one end of the tube, and the opposite extreme was closed with a sample holder beneath a rigid termination. The microphone signals were processed in real time by an FFT signal analyzer (Bruel & Kjaer Pulse C3560-C, Madrid, Spain), providing a broadband random signal as input to the loudspeaker. The measurement system was controlled by a notebook. The device set-up is shown in Figure 3. Three samples were measured to obtain each experimental value.

### 2.5. Measurements of Airflow Resistivity

Airflow resistivity is directly related to the capacity of the material to absorb sound energy, and it is defined as the resistance experienced by air as it passes through a material with a specific thickness (*d*). Airflow resistivity can be calculated as the quotient between the ratio of the difference in air pressure across the material relative to atmospheric pressure (Δ*p*), to the volumetric ratio of air flow through the material. This is mathematically expressed as [23]:

(3) $$\sigma =\frac{\Delta p}{Vd}$$

where *V* is the velocity of the airflow passing through the specimen cross-sectional area [24].

Airflow resistivity has been determined according to the standardized testing procedure described in ISO 9053-1:2020 [24]. This method is based on passing steady-state airflow through a sample. Annex A of this standard indicates that the airflow resistivity measurement can be estimated by measurements on impedance tubes. The indirect method of Ingard and Dear (1985) [25] has been validated by various studies as a valid procedure for estimating the airflow resistivity of porous materials. Using a cylindrical impedance tube with a rigid end, a sound source and two calibrated microphones placed before and after the sample, the value of the material’s air flow resistivity can be obtained.

**Figure 4:**
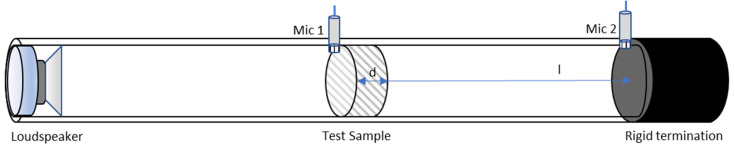
Scheme of the experimental set-up used to measure the airflow resistivity. Both microphones are mounted at different sides of the sample.

In this study, the Ingard and Dear method [25] was used to carry out tests of the sheep wool/SPI biocomposites with added coffee grounds. The test set-up diagram can be seen in Figure 4.

### 2.6. Empirical Model

The most common model to describe the acoustic behavior of fibrous materials is the one developed by Delany and Bazley (1970) [26]. Using this model, only the determination of the airflow resistivity (*σ*) of the porous material is necessary to calculate acoustic impedance. The Delany and Bazley relationships provide the following fits, in MKS units [26]:

(4) $$z_{c}=\rho c\left(1+c_{1}\chi^{-c_{2}}-jc_{3}\chi^{-c_{4}}\right)$$

(5) $$k_{c}=\frac{\omega }{c}\left(1+c_{5}\chi^{-c_{6}}-jc_{7}\chi^{-c_{8}}\right)$$

where *z_c_* is the characteristic wave impedance, *k_c_* is the characteristic sound propagation constant, *ρ* is the air density, *c* is the sound speed, *ω* is the angular frequency (*ω* = 2*πf*), *f* is the frequency, *C_i_* (*i* = 1,…,8) are eight numerical coefficients, and *χ* = *ρf*/*σ* is a dimensionless parameter [27]. These empirical formulae provide accurate results in the 0.01 < *χ* < 1.0 range [28].

The surface impedance (*z_s_*) is [18]:

(6) $$z_{s}=-jz_{c}\coth\left(k_{c}d\right)$$

where *d* is the thickness of the material.

The normal incidence sound absorption coefficient ($\ddot{\hat{\alpha }}$) is determined as [18]:

(7) $$\ddot{\hat{\alpha }}=1-{\left|\frac{z_{s}-\rho c}{z_{s}+\rho c}\right|}^{2}$$

To perform the adjustment, an error function that compares the estimated acoustic impedance with the measured one is programmed. A quadratic error function is constructed [18]:

(8) $$\varepsilon =\sum_{i=1}^{N}{\left(Z_{i}-{\hat{Z}}_{i}\right)}^{2}$$

where $Z_{i}$ is the impedance measured for a sample at the i-th frequency, and ${\hat{Z}}_{i}$ is the corresponding estimated value. To minimize the function, a genetic algorithm was programmed in MATLAB R2025a, which does not require initial iteration and allows to limit the range of the coefficients $C_{i}\left(i=1,\ldots ,8\right)$ to be obtained.

The genetic algorithm solves optimization problems with any type of constraints. It operates by generating an initial population and then creating successive generations through random mutations. In each generation, it selects the individuals that best fit the objective and uses them to produce new mutations. In the implementation, all coefficients to be adjusted are constrained between 0 and 1. The stopping criteria are either reaching a maximum of 200 generations or achieving a tolerance of 10^−6^. If the tolerance is not met, the algorithm returns the best result obtained from the 200 generations.

The eight coefficients in Equations (4) and (5) must be calculated specifically for each type of absorbing material, since each porous structure has its own characteristic features. Providing this, the Delany–Bazley model can be successfully applied to predict the sound absorbing behavior of different materials, including those based on natural fibers.

## 3. Results and Discussion

*TL* is a fundamental parameter to characterize sound insulation performance at in-lab measurements. It refers to the reduction in sound intensity as it passes through a barrier like a wall or partition. This is a measure of how well a material or structure blocks sound, and a higher *TL* indicates better soundproofing. In this sense, it is related to the material’s inherent sound-blocking capacity. To determine it, conditions in the measuring device tube are carefully designed to avoid transmissions that are not perpendicular to the tested sample, and thus axial to the direction of the tube.

Despite this, this special lab environment is not the case in real working performance of the material; when a sound-insulating material works in situ, it is impossible to subtract the effect of the surrounding sound transmissions, via adjacent walls or partitions, the so-called indirect paths. Thus, it is of paramount importance the way that the insulation material is mechanically coupled with its environment. Moreover, materials that are not inherently efficient at blocking sound can constitute powerful soundproofing structures, when combined with other materials with complementary mechanical and microstructural properties by means of appropriate couplings.

As a result, it is more proper to talk about insulating systems rather than about insulating materials, as materials never work alone on their own. Nevertheless, determination of *TL* is crucial to understanding the soundproofing capacity of a given material, and it is indispensable when characterizing it. For this reason, this analysis complements the acoustic characterization carried out in a previous publication [19] and is necessary to assess the viability of biocomposites with coffee grounds for acoustic application.

**Figure 5:**
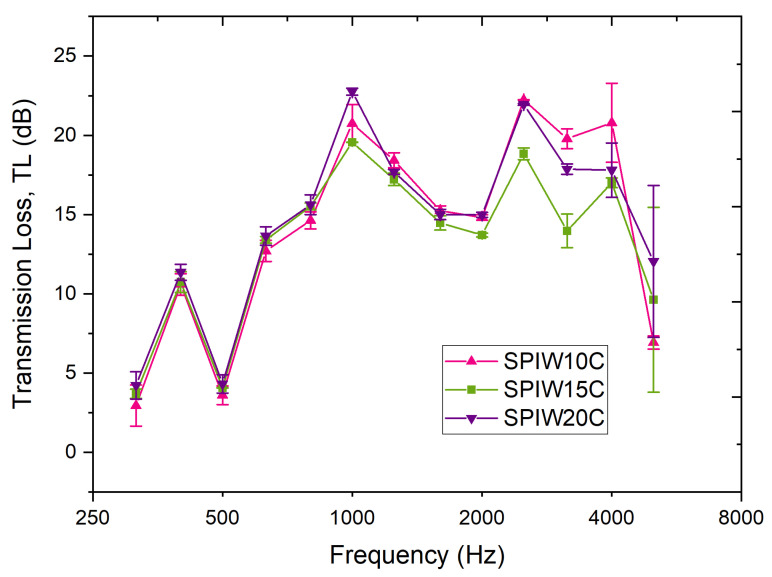
Transmission loss (*TL*) for the soy protein isolate (SPI) biocomposites with sheep wool (W) and spent coffee grounds (C), SPIW10C, SPIW15C, and SPIW20C biocomposites, as a function of frequency.

Obtained values for *TL* are given in Figure 5. Table A1 shows the ANOVA analysis of statistically significant differences among values of the same frequency. Curves show an overall increase in *TL* as the frequency increases, with a valley at 1250–2000 Hz and a sudden decrease above 4000 Hz. As the ANOVA analysis shows, the best biocomposites for sound insulation appear to be SPIW10C and SPIW20C, as they display the highest *TL* values, 22.24 ± 0.01 dB and 21.95 ± 0.06, respectively, at 2500 Hz, the typical frequencies of human speech and maximum sensitivity of human audition, with no significant difference between both values. Moreover, both biocomposites exhibit consistently high values around 19 dB at 3150 and 4000 Hz. Nevertheless, the significantly highest record is achieved by SPIW20C, with 22.8 ± 0.3 dB at 1000 Hz.

In addition, if the mean value of *TL* is calculated for all frequencies (as shown in Table 1), SPIW10C and SPIW20C are the best performing biocomposites overall, with values of 14.1 and 14.6 dB, respectively. It is remarkable that all the biocomposites have densities of 60–75 kg/cm^3^ (see Table 1), they do not show significant differences among them (lying within the error) and, thus, density does not seem to play a key role, explaining a better insulating behavior for SPIW20C. Biocomposites of this work show *TL* values that are even higher and, thus, better than *TL* values of commercial synthetic materials [29,30,31].

**Figure 6:**
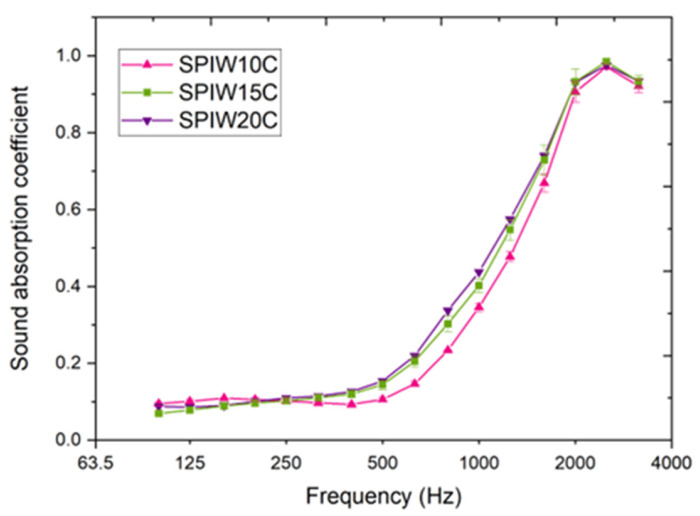
Sound absorption coefficient at normal incidence for the soy protein isolate (SPI) biocomposites with sheep wool (W) and spent coffee grounds (C), SPIW10C, SPIW15C, and SPIW20C biocomposites, as a function of frequency.

Results for the normal incidence sound absorption coefficient of the biocomposites SPIW10C, SPIW15C, and SPIW20C are shown as a function of frequency in Figure 6. Table A2 presents the ANOVA analysis of statistically significant differences among values of the same frequency.

The absorption coefficient curves observed exhibit the typical behavior of sound absorbing fibro-porous materials, showing low absorption at lower frequencies, which increases as the frequency rises. Regardless of the biocomposite composition, all samples demonstrate absorption coefficients approaching unity at higher frequencies, despite the low thickness of the samples at around 20 mm. Among the different samples, SPIW15C and SPIWC20 demonstrate the significantly highest values at the range of 630–800 Hz.

The shift in maximum frequency with composition can be associated with a quarter-wavelength resonance effect. In previous studies [32], mixtures were also analyzed and it was observed that the peak frequencies varied depending on the mixing ratio. The materials composing the samples cause variations in the speed of sound propagation through the medium. As the speed of sound changes, so does the quarter-wavelength resonance, resulting in a shift in the frequency peak. This effect was also observed in previous studies [33], where natural fibers were used as the base material.

The airflow resistance and airflow resistivity (airflow resistance/sample thickness) determined in this work are given in Table 1. In the Spanish legislation there is a requirement of a minimum airflow resistivity value for building materials to be applied; this value is required by the Building Technical Code (2006) [34]. The material catalogue linked to this Code specifies a minimum airflow resistivity of 5 kPa·s·m^−2^ for use in walls, floors, or roofs. Additionally, certain prediction expressions, which are only applicable when the airflow resistivity exceeds 5 kPa·s·m^−2^, are provided in the UNE-EN ISO 12354-1:2018 standard [35].

The maximum value obtained in this work is 18 ± 2 kPa s m^−2^ in the case of SPIW20C, and the lowest one, 14 ± 5 kPa s m^−2^ for SPIW10C. Thus, these values of airflow resistivity almost triple the required minimum, which underscores the potential of these biocomposites for constituting an alternative to synthetic materials. They also outperform the values of biocomposites without spent coffee grounds, namely 5.62 kPa s m^−2^ for SPIW10, 10.3 kPa s m^−2^ for SPIW15, and 11.6 kPa s m^−2^ for SPIW20 [18]. It is interesting to note that SPIW20C is the most efficient sound absorbing material studied in this work, as it shows the highest airflow resistivity and high sound absorption coefficient values at the 630–800 Hz range. Both parameters are related since the parameter that most significantly influences the increase in the absorption coefficient is airflow resistance, which is defined as the flow resistivity multiplied by the material thickness. The higher the airflow resistance, the greater the sound absorption, and the more it shifts toward lower frequencies. Theoretically, doubling this parameter would shift the absorption curve toward lower frequencies and generally increase the absorption coefficient. This behavior can be modeled using Equations (6) and (7). Porosity and tortuosity are also influential parameters, but as shown in the model, the most critical one is flow resistivity.

It is worth mentioning that good performance at sound absorption does not guarantee a good performance at inherent soundproofing, or vice versa; on the contrary, these properties are often in contradiction, and the optimal density ranges for the corresponding materials are different. In this context, it is remarkable to observe how SPIW20C displays the best records of the biocomposite series in both aspects. On the one hand, sound absorption capacity can be related with a fibro-porous microstructure with tortuous paths for sound transmission, which increase viscous losses and energy dissipation. This is the case for the biocomposites of this work. In this direction and within certain limits, increasing open porosity while decreasing volume density may increase sound absorption capacity, as sound encounters more surfaces at its propagation. In a previous work [19], porosities of the SPIWC series biocomposites were determined to be around 87–89%, but they did not show significant differences among them, and neither did volume density, as explained above. On the other hand, sound insulation typically requires higher volume densities for noise reduction, because the increased mass and stiffness make it harder for sound waves to pass through a structure. In this study, all the biocomposites analyzed, and particularly SPIW20C, seem to have achieved a good compromise, because measured values are relevant and competitive in both facets. As it has been mentioned, density values of the SPIWC biocomposites are around 62–72 kg/m^3^, and they can be considered medium densities for sound absorption (low: 45–60 kg/m^3^, and high: 80–140 kg/m^3^), but low densities for sound insulation (high: above 140 kg/m^3^). In comparison to the SPIW series, addition of coffee grounds increased volume density (SPIW10, 53.0 kg/m^3^; SPIW15, 54.2 kg/m^3^; and SPIW20, 61.2 kg/m^3^ [18]), but it seems that this increase has not cogged open porosity, as airflow resistivity and sound absorption are higher for the SPIWC biocomposites.

With the input of the measured airflow resistivity values, Equations (4) and (5) provided a model of sound absorption for each composition. Table 2 shows the results of the adjustment of the implemented genetic algorithms.

**Figure 7:**
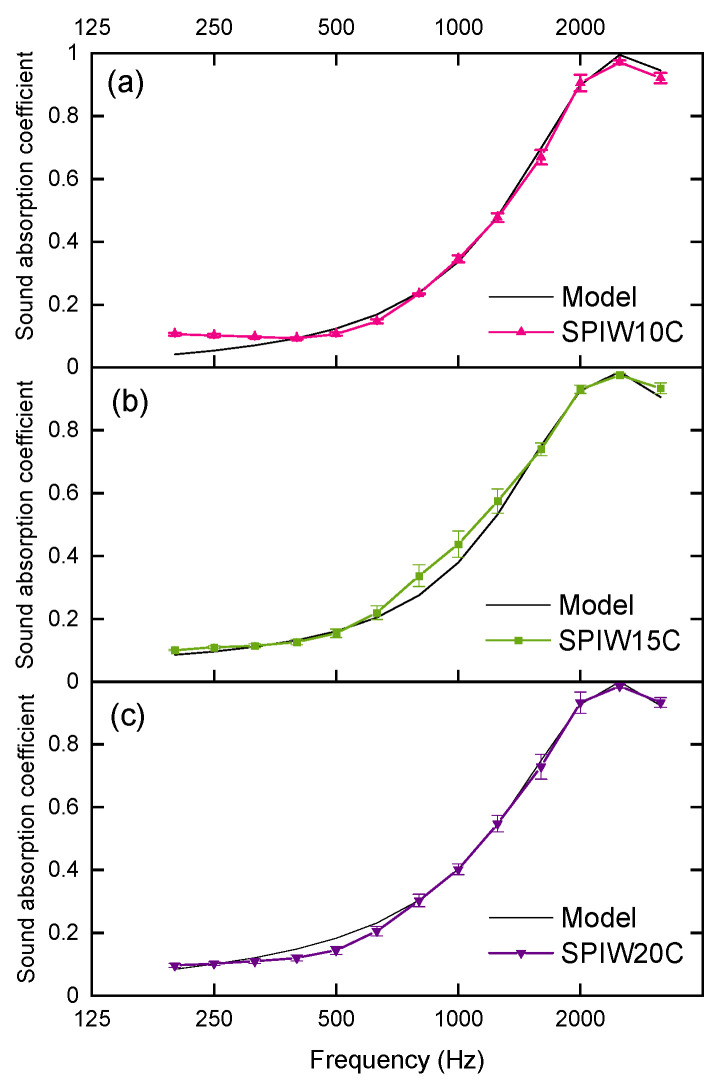
Sound absorption coefficients at normal incidence as a function of frequency for the soy protein isolate (SPI) biocomposites with sheep wool (W) and spent coffee grounds (C): (**a**) SPIW10C, (**b**) SPIW15C, and (**c**) SPIW20C.

Figure 7 presents the experimental results of the sound absorption coefficient at normal incidence as a function of frequency, along with the corresponding results obtained by the empirical model using the best-fitting coefficients from Table 2. Figure 7 depicts the adjustments of all samples, both in normalized acoustic impedance module and in the absorption coefficient at normal incidence. The model adequately adjusted the values for all samples and, therefore, it constitutes a useful predicting tool for the sound absorbing behavior of biocomposites. Similar genetic algorithms have been applied in the bibliography to predict sound absorption coefficients of natural absorbers. For example, Putra et al. (2021) [36] have studied kenaf fibers as a test material with good results, for a modeling method that can be applied for other natural fibers. In this case, the function of the genetic algorithm compared the experimental absorption coefficient and the predicted absorption coefficient calculated from the Johnson–Champoux–Allard (JCA) model. These authors have analyzed the sound absorption coefficient of natural durian husk fibers according to an analogous methodology [37].

## 4. Conclusions

In this work, sustainable biocomposites were prepared with the aim of valorizing bio-wastes such as sheep wool and spent coffee grounds. As far as *TL* and sound insulation are concerned, SPIW20C samples exhibited the best acoustic behavior. Furthermore, the obtained values were competitive and even better than those of commercial options. For all biocomposites, sound absorption coefficients rose as the frequency increased, and at high frequencies they almost reached one, which is the optimal top value. Additionally, airflow resistivity of sheep wool/soy protein biocomposites was determined experimentally and values almost triple the required minimum of 5 kPa s m^−2^. SPIW15C and SPIW20C exhibited the best sound absorption coefficient whereas airflow resistivity values were better for SPIW20C. The balanced compromise between good performance at sound insulation and at sound absorption is a key point of this work. Furthermore, an empirical model, developed based on airflow resistivity values, efficiently predicted sound absorption coefficients. Therefore, it can constitute a useful tool when designing acoustic solutions, and it can save energy and reduce costs in this process. Summing up, it can be concluded that SPIW20C is the most efficient biocomposite overall, confirming the potential of these sheep wool/SPI biocomposites as competitive sound absorbers in building construction industry, with the added value of their entirely renewable origin and environmentally sustainable approach.

## Figures and Tables

**Table 1:** Density, thickness, mean *TL* at all frequencies, airflow resistivity, and airflow resistance of SPIW10C, SPIW15C, and SPIW20C biocomposites.

Samples	Density (kg/m^3^)	Thickness (mm)	Mean *TL* (dB)	Airflow Resistivity (kPa s m^−2^)	Airflow Resistance (Pa s m^−1^)
SPIW10C	72 ± 8	16.9 ± 0.9	14.1	14 ± 5	200 ± 100
SPIW15C	62 ± 1	21.7 ± 0.4	13.2	15 ± 2	330 ± 50
SPIW20C	67 ± 3	18.8 ± 0.5	14.6	18 ± 2	330 ± 60

**Table 2:** Fitting coefficients in Equations (4) and (5) determined for SPIW10C, SPIW15C, and SPIW20C biocomposites.

Samples	C_1_	C_2_	C_3_	C_4_	C_5_	C_6_	C_7_	C_8_	*ε*
SPIW10C	0.8449	0.4074	0.0227	0.0098	0.4344	0.0602	0.4441	0.6054	1.8012
SPIW15C	0.5927	0.1071	0.2424	0.1297	0.5499	0.1220	0.3297	0.3919	1.5930
SPIW20C	0.4150	0.6861	0.1369	0.0260	0.2859	0.2873	0.1350	0.9966	2.1308
*	0.0571	0.7540	0.0870	0.7320	0.1890	0.5950	0.0978	0.7000	

* The coefficients of the Delany and Bazley formula are shown for comparison.

## Data Availability

The original contributions presented in this study are included in the article. Further inquiries can be directed to the corresponding authors.

## Appendix A

**Table A1:** Transmission loss values (*TL*) for soy protein isolate (SPI) biocomposites with a sheep wool content of 10, 15, and 20 wt%. (W). Mean values followed by the same letter at the same frequency do not differ significantly (*p* > 0.05) according to Tukey’s multiple range test.

	SPIW10C		SPIW15C		SPIW20C	
f (Hz)	Mean	SD	Mean	SD	Mean	SD
100	0.10 ^a^	0.01	0.09 ^a.b^	0.01	0.07 ^b^	0.00
125	0.10 ^a^	0.01	0.09 ^a.b^	0.00	0.08 ^b^	0.00
160	0.11 ^a^	0.00	0.09 ^b^	0.00	0.09 ^b^	0.01
200	0.11 ^a^	0.00	0.10 ^a^	0.00	0.10 ^a^	0.01
250	0.10 ^a^	0.00	0.11 ^a^	0.00	0.10 ^a^	0.01
315	0.10 ^a^	0.00	0.11 ^a^	0.01	0.11 ^a^	0.01
400	0.09 ^a^	0.00	0.13 ^a^	0.01	0.12 ^a^	0.01
500	0.11 ^a^	0.01	0.15 ^a^	0.01	0.14 ^a^	0.01
630	0.15 ^a^	0.01	0.22 ^b^	0.02	0.21 ^a.b^	0.02
800	0.23 ^a^	0.00	0.34 ^b^	0.03	0.30 ^a.b^	0.02
1000	0.35 ^a^	0.01	0.44 ^a^	0.04	0.40 ^a^	0.02
1250	0.48 ^a^	0.01	0.57 ^a^	0.04	0.55 ^a^	0.03
1600	0.67 ^a^	0.02	0.74 ^a^	0.02	0.73 ^a^	0.04
2000	0.91 ^a^	0.03	0.93 ^a^	0.01	0.93 ^a^	0.03
2500	0.97 ^a^	0.01	0.98 ^a^	0.01	0.99 ^a^	0.01
3150	0.92 ^a^	0.02	0.93 ^a^	0.02	0.93 ^a^	0.02

**Table A2:** Coefficient of absorption at normal incidence for soy protein isolate (SPI) biocomposites with a sheep wool content of 10, 15, and 20 wt%. (W). Mean values followed by the same letter at the same frequency do not differ significantly (*p* > 0.05) according to Tukey’s multiple range test.

	SPIW10C		SPIW15C		SPIW20C	
f (Hz)	Mean	SD	Mean	SD	Mean	SD
318.75	2.95 ^a^	1.30	3.70 ^a^	0.29	4.23 ^a^	0.86
400	10.59 ^a^	0.68	10.75 ^a^	0.66	11.37 ^a^	0.51
500	3.61 ^a^	0.59	4.07 ^a^	0.01	4.33 ^a^	0.58
630	12.71 ^a^	0.67	13.41 ^a^	0.21	13.65 ^a^	0.58
800	14.64 ^a^	0.54	15.52 ^a^	0.22	15.62 ^a^	0.62
1000	20.75 ^a.b^	1.19	19.56 ^a^	0.02	22.76 ^b^	0.22
1250	18.43 ^a^	0.47	17.22 ^b^	0.39	17.69 ^a.b^	0.16
1600	15.24 ^a^	0.31	14.48 ^a^	0.45	15.00 ^a^	0.32
2000	14.82 ^a^	0.16	13.71 ^b^	0.12	14.99 ^a^	0.16
2500	22.24 ^a^	0.01	18.84 ^b^	0.38	21.95 ^a^	0.06
3150	19.79 ^a^	0.63	13.98 ^b^	1.06	17.87 ^a^	0.33
4000	20.80 ^a^	2.50	17.01 ^a^	0.31	17.81 ^a^	1.72
5000	6.93 ^a^	0.41	9.62 ^a^	5.83	12.05 ^a^	4.79
